# Fournier’s gangrene in female patient with Turner’s syndrome: A case report

**DOI:** 10.1016/j.eucr.2022.102137

**Published:** 2022-06-17

**Authors:** Jupiter Sibarani, Alwin Soetandar

**Affiliations:** Department of Urology, Hasan Sadikin Academic Medical Center, Faculty of Medicine, Universitas Padjadjaran Bandung, Indonesia

**Keywords:** Fournier's gangrene, Vulvar synechiae, Turner's syndrome

## Abstract

This case reported Fournier's Gangrene (FG) case in 22 year old woman, which is rarely found in young woman, with Turner's Syndrome, vulvar synechiae, uterine agenesis, and voiding disorders. Turner's Syndrome can lead to vulvar synechiae and voiding disorders. This condition is exacerbated by *E. coli* bacterial infection, causes tissue necrosis and leads to FG. Urgent synechiae incision and necrotomy debridement were successfully performed to remove the necrotic tissue followed by graft closure and flap reconstruction. Rapid administration of broad-spectrum antibiotics is also important. These treatments are considered as the key of successful outcomes of FG.

## 1 Introduction

The incidence of FG among females tends to be rare. The male to female ratio was reported at 10:1.^1^^,^^2^ Most men and women with FG will have both genital and perineal involvement. Women will almost always have vulvar or labial involvement (95%–100%). Although typically affects men, we present here the FG, a rare disease, found in a young woman with Turner Syndrome (TS).

## 2 Case description

A 22-year-old woman has had a chief complaint of pain and swelling in the vulva region since a week before admission. Swelling is getting larger in the suprapubic and left flank areas. The complaints are accompanied by redness in the area. She had a history of intermittent fever since 5 days ago. She had a history of amenorrhea and delayed secondary sexual development and was diagnosed with uterine agenesis 5 years ago. She had a history of difficulty urinating since 6 months ago accompanied by straining during urination, and nocturia more than 3 times per night which become worsened a month before admission. Other complaints of urination are incomplete emptying and a weak urinary stream. Daily urine production about 1000 cc/24 hours, yellowish. Previously, she had already undergone cystoscopy, urethral dilatation, and synechia incision in another hospital.

Physical examination showed urological state in the form of tenderness, redness, swelling, and fluctuation at right and left flank region. Redness, swelling, and fluctuation were found at suprapubic and vulva region.

Further examination with KUB Ultrasound showed normal limit. Flank ultrasound showed thickening of cutis and sub cutis tissue.

From the laboratory results, the hemoglobin, thrombocyte, and albumin were low (8,2 g/dl, 25.000/μL, and 1.5 mg/dl) and other laboratory parameters are within normal limits. Urinalysis showed microscopic hematuria (20–28/hpf) and leukocyturia (20–29/hpf). Moreover, culture examination showed a positive result for *Escherichia coli*. The patient was subsequently diagnosed with vulvar synechiae which was the cause of the urinoma and progressed to FG. The result of Fournier's Gangrene Scoring Index (FGSI) is 10. All these findings lead to the diagnosis of FG with vulvar synechiae and uterine agenesis.

**Fig. 1:**
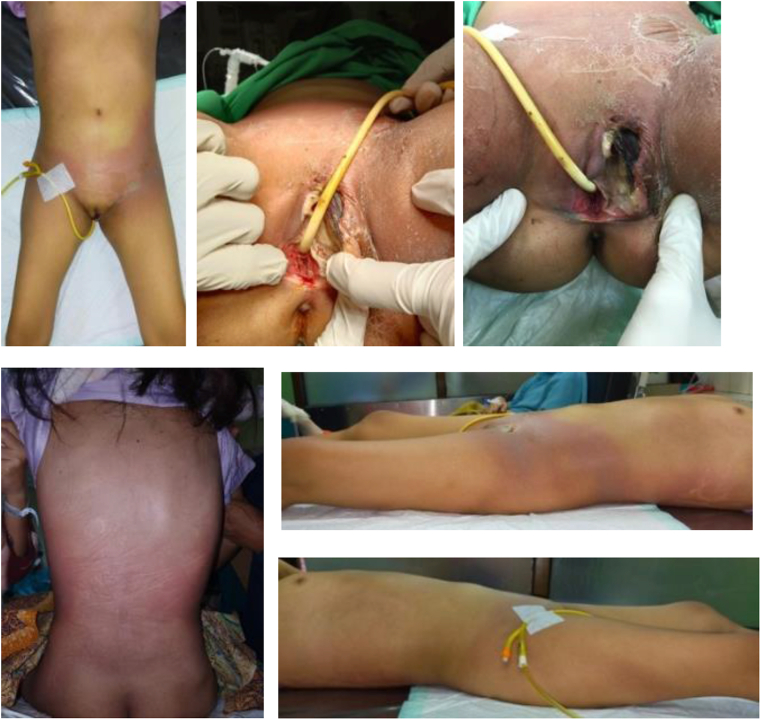
Preoperative necrotomy debridement (september 12th, 2021).

**Fig. 2:**
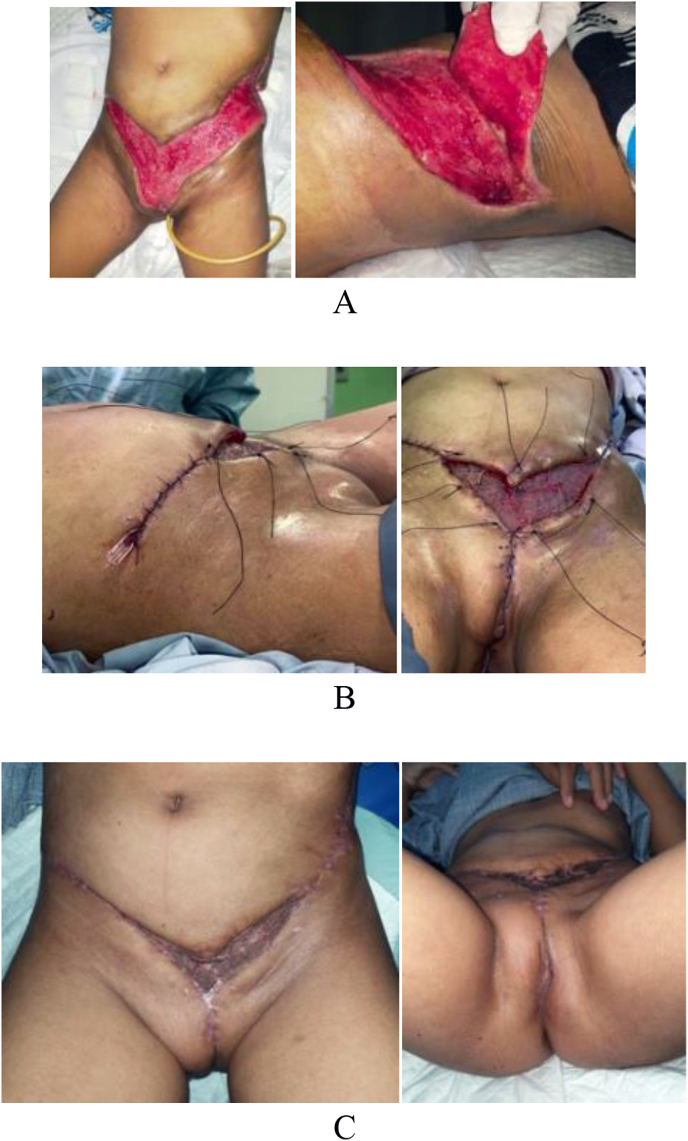
Post-operative STSG and advancement flap. A. Postoperative Necrotomy Debridement (October 4th, 2021). B. Postoperative STSG and Advancement Flap (October 6th, 2021). C. Follow Up 4 Months Postoperative (November 22nd, 2021).

The patient was treated with Meropenem 3 × 1 gram, Omeprazole 2 × 40 mg, Paracetamol 3 × 500 mg, VipAlbumin® 3 × 2 tablets, and HP Pro 3 × 1 tablet. The patient underwent an urgent synechial incision followed by necrotomy debridement in Hasan Sadikin Hospital by a urologist. Clinical pictures of pre-operative necrotomy debridement are shown in Fig. 1. Necrotomy debridement was successfully carried out. The patient also underwent graft skin closure and flapping surgery on October 6, 2021, by a plastic surgeon. The remaining raw surface after debridement was covered with STSG. Undermining was also performed on the edges of the wound and then an advance flap on some of the defects (Fig. 2). The patient should rub olive oil on the epithelialized area, raw surface with burnazin plus ointment, and should do the bath education. Obstetrics & gynecology re-consultation is needed for the evaluation of internal reproductive organs and vaginal reconstruction plan.

**Fig. 3:**
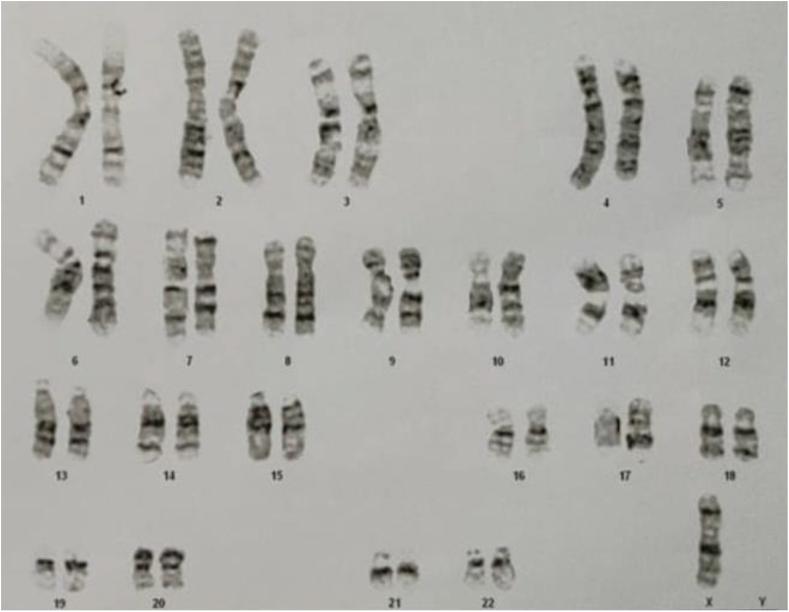
Chromosomal analysis result.

After carrying out a chromosomal examination, it was found that the patient has only one X chromosome (45, X0) (Fig. 3), so the patient was diagnosed with TS. It is supported by the laboratory results that showed low estrogen level (47.49 IU/ml), high FSH (187.78 IU/ml), and LH level (47.49 IU/ml) respectively. Meanwhile, estrogen levels are low (12.2 pg/ml).

## 3 Discussion

TS is a chromosomal condition in women in which one X chromosome is absent. Multiple recurrent infections in TS could cause a chronic inflammation such as vulvar synechiae. This condition in adults can include urinary problems such as urinary retention and dyspareunia.^3^ In this patient, vulvar synechiae was found which was suspected to be related to her TS condition. Synechiae can cause voiding disorders so that it can have an impact on urinary retention and a weak urinary stream, such as found in this case.

FG is a rapidly progressive necrotizing infection of the perineal, genital, or anorectal region. The etiologic origin of FG includes dermatologic infections that are usually caused mostly by *E. coli*, streptococcal species, and *P. aeruginosa*. In this case, the patient's tissue culture examination showed a positive result of *E. coli*. This finding also strengthens the notion that the urinary retention and urinoma experienced by the patient has an infection. As a result, the condition triggers cell necrosis which causes FG. In the treatment of infection in FG, it is necessary to start effective broad-spectrum effective earlier.^4^

The typical signs of FG are pain, swelling, and erythema of the perineum and genitalia. This is in accordance with the patient's complaints in the form of pain, swelling, and redness at vulva region since a week before admission. FGSI scoring systems are valuable for predicting mortality in patients with FG. This patient had an FGSI score of 10. An FGSI score of more than 9 could increase the risk of motility up to 75%.^1^

This patient took meropenem which antibiotics used for FG case. The cornerstones of treatment of Fournier’s gangrene are urgent surgical debridement of all necrotic tissue as well as high doses of broad-spectrum antibiotics. This is in line with the surgical management performed in this case. The patient also underwent reconstructive surgery, the goal is shifted to cover surgical wounds with the best functional and cosmetic results possible as well as minimal morbidity and mortality and graft closure is the main reconstruction option.^5^

## 4 Conclusion

Fournier’s Gangrene is rare in women. In this case, a female patient with Turner Syndrome had vulvar synechiae and voiding disorders. This condition is exacerbated by the presence of *E. coli* bacterial infection which causes tissue necrosis and leads to Fournier's Gangrene.

## Declaration of competing interest

Authors declare no conflict of interest.
